# Adolescent self-report and parent proxy-report of health-related quality of life: an analysis of validity and reliability of PedsQL™ 4.0 among a sample of Malaysian adolescents and their parents

**DOI:** 10.1186/s12955-015-0234-4

**Published:** 2015-04-08

**Authors:** Sanker Kaartina, Yit Siew Chin, Rezali Fara Wahida, Fui Chee Woon, Chu Chien Hiew, Mohd Shariff Zalilah, Mohd Taib Mohd Nasir

**Affiliations:** Department of Nutrition and Dietetics, Faculty of Medicine and Health Sciences, Universiti Putra Malaysia, Serdang Selangor Darul Ehsan, 43400 Kuala Lumpur, Malaysia; Research Centre of Excellence, Nutrition and Non-communicable Diseases, Faculty of Medicine and Health Sciences, Universiti Putra Malaysia, Kuala Lumpur, Malaysia

**Keywords:** Health-related quality of life, Validity, Reliability, Adolescents, Parents, Non-clinical

## Abstract

**Background:**

The Pediatric Quality of Life Inventory™ Generic Core Scales (PedsQL™) 4.0 is a generalized assessment of health-related quality of life (HRQoL) based on adolescent self-report and parent proxy-report. This study aims to determine the construct validity and reliability of PedsQL™ 4.0 among a sample of Malaysian adolescents and parents.

**Methods:**

A cross-sectional study was carried out at three selected public schools in the state of Selangor. A total of 379 Malaysian adolescents completed the PedsQL™ 4.0 adolescent self-report and 218 (55.9%) parents completed the PedsQL™ 4.0 parent proxy-report. Weight and height of adolescents were measured and BMI-for-age by sex was used to determine their body weight status.

**Results:**

There were 50.8% male and 49.2% female adolescents who participated in this study (14.25 ± 1.23 years). The prevalence of overweight and obesity (25.8%) was four times higher than the prevalence of severe thinness and thinness (6.1%). Construct validity was analyzed using Confirmatory Factor Analysis (CFA). Based on CFA, adolescent self-report and parent proxy-report met the criteria of convergent validity (factor loading > 0.5, Average Variance Extracted (AVE) > 0.5, Construct Reliability > 0.7) and showed good fit to the data. The adolescent self-report and parent proxy-report exhibited discriminant validity as the AVE values were larger than the R^2^ values. Cronbach’s alpha coefficients of the adolescent self-report (α = 0.862) and parent proxy-report (α = 0.922) showed these instruments are reliable. Parents perceived the HRQoL of adolescents was poorer compared to the perception of the adolescent themselves (t = 5.92, p < 0.01). There was no significant difference in total HRQoL score between male and female adolescents (t = 0.858, p > 0.05). Parent proxy-report was negatively associated with the adolescents’ BMI-for-age (r = -0.152, p < 0.05) whereas no significant association was found between adolescent self-report and BMI-for-age (r = 0.001, p > 0.05).

**Conclusion:**

Adolescent self-report and parent proxy-report of the PedsQL™ 4.0 are valid and reliable to assess HRQoL of Malaysian adolescents. Future studies are recommended to use both adolescent self-report and parent-proxy report of HRQoL as adolescents and parents can provide different perspectives on HRQoL of adolescents.

## Background

The WHO Quality of Life Group [1] defines health-related quality of life (HRQoL) as an individual’s perception of their place in life comprising the culture and value system of which they live in and their reactions towards goals, standards and concern. HRQoL is a broad range of concepts that explains how quality of life affects an individual’s physical health, psychological state, independence, social relationships and his perception towards environmental influences [1,2]. Evaluation of HRQoL is mainly based on the subjective perception of the physical, emotional, social functioning as well as the overall well-being of an individual [3]. As delineated by the WHO [4,5], a HRQoL instrument must be multidimensional, comprising at least a minimum of three dimensions, namely physical, psychological (emotional and cognitive) and social health dimensions.

The physical dimension of HRQoL measures the changes in physical activities carried out by individuals daily. The emotional dimension of HRQoL assesses the satisfaction, achievement of personal goals, personal control, social interaction, self-concept and self esteem [6], whereas the social functioning dimension of HRQoL assesses the existence of social relationships and activities [3,7]. In addition, a school functioning dimension was later included in HRQoL to evaluate the frequency of a child absent from school due to illness and admitted to the hospital [8]. Although not all ill children are hospitalized, school functioning is added to evaluate the HRQoL of hospitalized children as their HRQoL may be affected [8]. A HRQoL instrument should be brief and comprehensive, has good psychometric quality and should include both self- report and parent-proxy versions [9].

There is a need to assess the HRQoL in a general population as poor HRQoL may not only be caused by the presence of a disease, but could also be due to factors such as stigmatization [10], physical inabilities and poor psychological well-being [11]. The Pediatric Quality of Life Inventory™ 4.0 is recognized as one of the most frequently used HRQoL instruments to assess HRQoL [12]. This questionnaire, developed in the United States, consists of generic modules which can be used to compare between health conditions and certain disease specific modules such as cancer, diabetes and asthma which can be used to detect specific treatment effects. Furthermore, a parallel parent’s version of the PedsQL™ 4.0 questionnaire was developed to focus on a parent’s perception towards his/her child’s HRQoL [12,13].

The PedsQL™ 4.0 was previously used to measure the HRQoL of thalassaemia [14] and disabled Malaysian children [15]. It has been reported that HRQoL of obese adolescents in a non-clinical setting was similar to that of adolescents diagnosed with cancer [16]. However, to our knowledge, the HRQoL of Malaysian adolescents in a non-clinical population has never been assessed. Hence, there is a need to validate the questionnaire to enable the assessment of the HRQoL of Malaysian adolescents in a non-clinical setting. This study was carried out to determine the construct validity comprising convergent and discriminant validity, and the internal consistency of both the adolescent self- report and parent-proxy report of PedsQL™ 4.0 among Malaysian adolescents and their parents.

## Methodology

This cross-sectional study was conducted from 25 June to 19 July 2011 among secondary school students in the districts of Kajang and Bangi, state of Selangor, Malaysia. Three schools which met the inclusion criteria of being co-educational, multiracial in composition, non-religious and government public schools were selected for this study. Students in the examination school year (Form 3 and Form 5) were excluded from this study as instructed by the Ministry of Education.

Ethics approval were obtained from the Medical Research Ethics Committee of the Faculty of Medicine and Health Sciences, Universiti Putra Malaysia (UPM/FPSK/PADS/T7-MJKEtikaPer/F01 (JPD(U)_SEPT (10)38). Also, approval to conduct the study in government public schools was obtained from the Ministry of Education Malaysia, Selangor State Department of Education and the schools involved. Four trained researchers were involved during data collection. Their training focused on techniques of conducting anthropometric measurements and providing a standardized flow of instructions and procedures to the adolescents involved in the study. Any question by the adolescents on the questionnaire was answered by the project leader. Information sheets on the study and consent forms were distributed to the adolescents and their parents prior to data collection. Signed informed consent forms were collected on the day of data collection. A total of 390 adolescents and their parents were initially invited to participate in this study. There were 379 (97.2%) adolescents who consented and completed the self-report of PedsQL™ 4.0 whereas 218 (55.9%) parents returned the parent proxy-report of PedsQL™ 4.0.

### Measures

#### Socio-demographic background

Socio-demographic information including sex, ethnicity, age and date of birth were obtained from the adolescents. Education level and monthly income of parents were collected from the parents.

#### Anthropometric measurements

Body weight of adolescents was measured using a TANITA Digital Weighing Scale (HD 319) to the nearest 0.1 kg whereas a SECA body meter (SE 206) was used to measure height to the nearest 0.1 cm. Body weight status of adolescents was classified based on BMI-for-age by sex using the WHO Growth Reference (Severe thinness: <-3SD, Thinness: <-2SD, Normal weight: ≤-2SD to ≥1SD, Overweight: > + 1SD, Obesity: > + 2SD) [17].

#### Pediatric quality of life inventory™ Version 4.0 (PedsQL™ 4.0)

The PedsQL™ 4.0 version 13-18 years was used in this study. It is a generic tool that assesses the HRQoL of patients or healthy population among children and adolescents [18]. This 23-item PedsQL™ 4.0 consists of a parallel child/adolescent self-report and a parent proxy-report. Both the PedsQL™ 4.0 of adolescent self-report and parent proxy-report were reported to have high internal consistency and construct validity in a study conducted among 8591 children aged between 5 to 16 years in California [19].

The PedsQL™ 4.0 can be interpreted based on a scale order, lower and higher order. The lower order scale encompasses four dimensions [20]: (1) Physical Functioning (eight items), (2) Emotional Functioning (five items), (3) Social Functioning (five items) and (4) School Functioning (five items). These four dimensions (lower order of PedsQL™ 4.0) can be combined into two dimensions of a higher order scale. The higher order scale was derived due to the correlations observed among the four dimensions [12] whereby the physical functioning dimension was discriminated from the other three dimensions [21,22]. Specifically, a Psychosocial Health dimension encompassing emotional functioning, social functioning and school functioning dimensions, and a Physical Health dimension encompassing the physical functioning dimension [20] were derived in the higher order scale.

The PedsQL™ 4.0 requires the adolescent and parent to recall the frequency of problems which occurred with the adolescent in the past one month. A five-point response Likert scale (0 = never a problem; 1 = almost never a problem; 2 = sometimes a problem; 3 = often a problem; 4 = almost always a problem) was used for all items. All the items were then reverse scored and transformed into a 0-100 point scale (0 = 100; 1 = 75; 2 = 50; 3 = 25; 4 = 0), whereby a higher total score of PedsQL™ 4.0 indicates a better HRQoL of the adolescent.

The adolescent self-report and parent-proxy report of PedsQ™ 4.0 were translated from English to the Malay language by two postgraduate students with backgrounds in the Health Sciences fluent in both languages. The questionnaire was then back-translated into English by another two bi-lingual postgraduate students also with backgrounds in the Health Sciences. The English back-translated version was consistent with the original PedsQ™ 4.0 adolescent self-report and parent proxy-report.

### Statistical analysis

Data were analyzed using SPSS version 19. Missing values were replaced with the mean values of the items of the respective scale per individual respondent. Internal consistency which is defined as the extent to which all items of a test measure the same latent variable [8] was determined using Cronbach’s alpha coefficient (α). A Cronbach’s alpha coefficient (α) of at least 0.7 was considered as acceptable [8]. Pearson Product-Moment Correlation was used to determine the association between BMI-for-age and HRQoL of the adolescents.

Confirmatory factor analysis (CFA) was carried out using the Analysis of Moment Structure (AMOS) software version 19 to assess construct validity. Construct validity is a set of measured items which reflects the theoretical latent construct those items are designed to measure [23,24]. In this study, construct validity comprises convergent and discriminant validity, of the PedsQL™ 4.0. Convergent validity refers to a set of variables (items) that presume to measure a construct [25] which was tested using three indicators. The first indicator is the factor loadings of the items in the PedsQL™ 4.0. High factor loading (≥0.5) of items show high convergent validity of the items [26]. Secondly, average variance extracted (AVE) of the construct was determined. An instrument is valid when the AVE is greater than 0.5 indicating high convergent validity of the PedsQL™ 4.0 construct [27]. Thirdly, construct reliability (CR) of the model was determined whereby values between 0.6 and 0.7 can be accepted providing other indicators are good [28].

As part of convergent validity, the model fit of the instrument was tested. A number of fit indices were used to test the model fit: goodness-of-fit index (GFI), adjusted goodness-of-fit index (AGFI), comparative fit index (CFI), normed fit index (NFI), Tucker Lewis index (TLI) and root mean square error of approximation (RMSEA). Model fit was considered acceptable when the values of GFI, CFI, TLI, AGFI and NFI were above 0.9 and RMSEA less than 0.8 [25]. Any four of the fit indices within the acceptable range are sufficient to determine the model fit [25]. In addition, the Likelihood Ratio Test or chi-square difference test (CMIN/df) was used to determine the model adequacy whereby an acceptable range is less than five [25]. This test indicates the amount of difference between the expected and observed covariance matrices [28].

Discriminant validity refers to a set of variables presumed to measure different dimensions [28]. Discriminant validity was determined when the AVE of each construct is greater than the squared correlations between the constructs in the model [28]. Squared correlation (R^2^) was used as this indicates the items of each dimension belong to the construct they are associated with rather than the other constructs.

## Results

### Socio-demographic background

A total of 379 adolescents (50.8% males, 49.2% females), with a mean age of 14.25 ± 1.23 years, from three public schools in the state of Selangor participated in this study (Table 1). A majority of the respondents were Malay (68.1%), 17.2% were Chinese, 12.9% Indian and 1.8% were of other ethnic groups. Most of the adolescents were of normal weight (68.1%), but the combined percentage of overweight (14.2%) and obese (11.6%) adolescents was more than four times higher than the combined percentage of adolescents who were severely thin (1.6%) and thin (4.5%). Close to half of their parents (father: 40.3%; mother: 45.4%) have acquired a minimum of secondary school education. The parents also had a mean monthly income of RM 4364.40 ± 9444.15 with a minimum of RM 700.00 and maximum of RM 5000.00.

**Table 1:** **Socio-demographic background of adolescents (n = 379)**

**Background**	**Total (n = 379)**	**Male (n = 191)**	**Female (n = 188)**	***X*** ^**2**^ **value**
**Ethnicity**				2.11
**Malay**	258 (68.1)	133 (69.6)	125 (66.5)	
**Chinese**	65 (17.2)	29 (15.2)	36 (19.1)	
**Indian**	49 (12.9)	25 (13.1)	27 (12.8)	
**Others**	7 (1.8)	4 (2.1)	3 (1.6)	
**Age**				2.36
**13-14**	265 (69.9)	134 (70.1)	131 (69.7)	
**16-17**	114 (30.1)	57 (29.9)	57 (30.3)	
**Mean ± SD**	14.25 ± 1.23	14.26 ± 1.25	14.28 ± 1.23	
**Weight (kg)**				
**Mean ± SD**	51.9 ± 13.8	54.1 ± 15.1	50.0 ± 12.1	
**Height (cm)**				
**Mean ± SD**	158.3 ± 8.5	162.0 ± 9.1	154.6 ± 5.8	
**Body weight status**				2.12
**Severe thinness (<-3SD)**	6 (1.6)	3 (1.6)	3 (1.6)	
**Thinness (<-2SD)**	17 (4.5)	10 (5.2)	7 (3.7)	
**Normal weight (≤-2SD to ≥ +1SD)**	258 (68.1)	127 (66.5)	131 (69.7)	
**Overweight (> + 1SD)**	54 (14.2)	26 (13.6)	28 (14.9)	
**Obese (> + 2SD)**	44 (11.6)	25 (13.1)	19 (10.1)	
**Mean ± SD (z-score)**	0.09 ± 1.40	0.06 ± 1.52	0.13 ± 1.28	
**Father’s educational attainment (n = 218)**				10.57
**University**	52 (23.9)	24 (25.5)	28 (22.6)	
**STPM/Diploma/A level**	60 (27.5)	23 (24.5)	37 (29.8)	
**Secondary school**	88 (40.3)	43 (45.7)	45 (36.3)	
**Primary school**	15 (6.9)	4 (4.3)	11 (8.9)	
**No formal education**	3 (1.4)	0 (0.0)	3 (2.4)	
**Mother’s educational attainment (n = 218)**				8.32
**University**	41 (18.8)	17 (18.1)	24 (19.4)	
**STPM/Diploma/A level**	48 (22.0)	17 (18.1)	31 (25.0)	
**Secondary school**	99 (45.4)	51 (54.2)	48 (38.7)	
**Primary school**	20 (9.2)	5 (5.3)	15 (12.1)	
**No formal education**	10 (4.6)	4 (4.3)	6 (4.8)	
**Monthly parental income (RM) (n = 218)**	4364.40 ± 9444.15	3928.66 ± 3712.78	4768.81 ± 12624.85	

Note. Data presented are expressed as n (%).

### Validity

Construct validity which comprises convergent and discriminant validity was determined through CFA. Firstly, convergent validity was tested by examining the factor loadings of the items, AVE and CR. Based on CFA, factor loadings were determined for all 23 items of the PedsQL™ 4.0 adolescent self-report and parent proxy-report to decide whether to add or remove the items. As shown in Table 2, standardized factor loadings for the lower-order scale of the PedsQL™ 4.0 exceeded 0.5 for both the adolescent self-report and parent proxy-report indicating high convergent validity [26]. Standardized factor loadings for the higher-order scale of the PedsQL™ 4.0 for both the adolescent self-report and parent proxy-report also exceeded 0.5 with the highest value of 0.96 and lowest value of 0.54.

**Table 2:** **Summary of CFA & reliability results for PedsQL™** **4.0**

	**Cronbach coefficient α**	**Range of factor loadings**	**AVE**	**Construct reliability (CR)**	**CFI**	**NFI**	**TLI**	**RMSEA**	**CMIN/df**	**Convergent validity**	**Discriminant validity**
**PedsQL adolescent self-report Lower order**											
Physical function	0.861	0.54-0.84	0.564	0.871	0.938	0.927	0.909	0.06	1.280	Valid	Valid
Emotional function	0.817	0.53-0.84	0.588	0.823				0.06	4.527	Valid	Valid
Social function	0.850	0.66-0.83	0.539	0.853	0.965	0.958	0.896	0.08	3.868	Valid	Valid
School function	0.750	0.58-0.69	0.599	0.768				0.08	4.380	Valid	Valid
**Higher order**					0.981	0.975	0.943				
Physical health					0.903	0.941	0.860				
Psychosocial health	0.861	0.54-0.84	0.564	0.871				0.06	1.280	Valid	Valid
	0.890	0.53-0.87	0.698	0.827				0.08	3.891	Valid	Valid
					0.838	0.827	0.709				
					0.903	0.821	0.930				
**PedsQLparent proxy-report Lower order**											
Physical function	0.901	0.80-0.96	0.810	0.972	0.947	0.943	0.905	0.06	11.517	Valid	Valid
Emotional function	0.810	0.86-0.92	0.800	0.952				0.08	4.115	Valid	Valid
Social function	0.722	0.82-0.88	0.723	0.929	0.984	0.981	0.951	0.04	65.320	Valid	Valid
School function	0.775	0.77-0.96	0.797	0.951				0.06	22.356	Valid	Valid
**Higher order**					0.913	0.912	0.939				
Physical health	0.901	0.80-0.96	0.810	0.972				0.06	11.517	Valid	Valid
Psychosocial health	0.949	0.77-0.96	0.624	0.899	0.972	0.971	0.972	0.07	11.951	Valid	Valid
					0.947	0.43	0.905				
					0.932	0.94	0.911				

Note: CMIN/df- chi-square/ degrees of freedom, CFI- Comparative Fit Index, NFI- Normed Fit Index, TLI- Tucker Lewis Index, AVE- Average Variance Extracted, RMSEA- Root Mean Squared Error of Approximation.

Next, the AVE of the PedsQL™ 4.0 was tested. AVE is the mean variance extracted for the item loadings of a construct [29]. The AVE values for the lower and higher-order scale of the PedsQL™ 4.0 adolescent self-report and parent-proxy report were more than 0.5, indicating high convergent validity (Table 2). The AVE values for both adolescent self-report and parent proxy-report show that items in each dimension belong to their respective dimension [27].

Thirdly, dimensions in the PedsQL™ 4.0 met the construct reliability (CR) criterion as it is within the acceptable range (Construct Reliability (CR) ≥ 0.7). Specifically, CR of all items in the lower-order scale of the PedsQL™ 4.0 ranged from 0.768 to 0.853 for the adolescent self-report and 0.929 to 0.972 for the parent proxy-report (Table 2). As for the higher-order scale, the CR values ranged from 0.827 to 0.871 and 0.899 to 0.972 in both adolescent self-report and parent proxy-report, respectively.

Subsequently, model fit of the PedsQL™ 4.0 adolescent self-report and parent proxy-report was tested. A number of fit indices were used to determine the construct of PedsQL™ 4.0. The fit indices including CFI (>0.9), GFI (>0.9), NFI (>0.9), TLI (>0.9), AGFI (>0.9), RMSEA (<0.8) were used to determine model fit for both lower and higher-order scale of the PedsQL™ 4.0. Of the fit indices mentioned, four fit indices which were within the acceptable range were chosen to determine the model fit of the data [25]. Data of the fit indices in Table 2 demonstrate that the lower and higher-order scales of the PedsQL™ 4.0 adolescent self-report and parent proxy-report have good-to-excellent fit within the indices mentioned above.

In addition, the non-significance of chi-square goodness-of-fit (CMIN/df) demonstrates a good model fit. The recommended value for CMIN/df is less than 5.0 [29]. The CMIN/df obtained for the adolescent self-report ranged from 1.280 to 4.527 in the lower-order scale and the CMIN/df of the higher-order scale ranged from 1.280 to 3.891, which was within the acceptable range (Table 2). In the lower-order scale, CMIN/df value for the emotional functioning dimension was the dimension within the recommendable value for the parent proxy-report. The CMIN/df values for the physical functioning, social functioning and school functioning dimensions of the parent proxy-report were not within the acceptable range (>5.0). As for the higher-order scale of parent proxy-report, psychosocial health and physical health dimensions did not meet the recommended value (>5.0). However, lower and higher-order scales of the parent proxy-report had an adequate fit to the data when taking other fit indices into consideration (CFI, NFI, TLI, RMSEA). Therefore, both the adolescent self-report and parent proxy-report have an adequate fit to the data indicating that the PedsQL™ 4.0 model fit to the current data.

Further, discriminant validity was determined for the lower and higher-order scales of the PedsQL™ 4.0. In this study, discriminant validity was determined by comparing the AVE values of the dimensions with the squared correlation between the dimensions (R^2^). A larger AVE value when compared to the squared correlation between the dimensions (R^2^) would provide evidence of discriminant validity. The correlation between the lower and higher-order scale of the PedsQL™ 4.0 adolescent self-report ranged from 0.35 to 0.66. Thus, a maximum R^2^ value derived from this correlation was 0.44. Table 2 shows that the AVE values for the lower and higher-order scale of the PedsQL™ 4.0 adolescent self-report was more than 0.44 which provide evidence of discriminant validity. Also, the correlation between the lower and higher-order of the PedsQL™ 4.0 parent proxy-report in this study ranged from 0.33 to 0.56 which provides a maximum R^2^ of 0.31. As the AVEs were more than 0.31, the constructs exhibit sufficient discriminant validity for the parent proxy-report of the PedsQL™ 4.0.

### Reliability

Cronbach’s alpha coefficients (α) across the dimensions of the PedsQL™ 4.0 for the adolescent self-report and parent proxy-report are presented in Table 2. The Cronbach’s alpha coefficients of each dimension in the PedsQL™ 4.0 for both adolescent self-report and parent-proxy report were greater than 0.7 [26]. As shown in Table 2, the Cronbach’s alpha coefficients in the adolescent self-report were high (≥0.8) for both the lower-order and higher-order scales of the PedsQL™ 4.0. Also, the parent proxy-report for the higher-order scale of the PedsQL™ 4.0 was very high (≥0.9), while the Cronbach’s alpha coefficient for the lower-order scales were within the acceptable range (>0.7).

*Distribution of HRQoL scores in adolescents according to adolescent self-report and parent proxy-report.*

The descriptive statistics of adolescent HRQoL scores according to adolescent self-report and parent proxy-report are presented in Table 3. Parents perceived their children to have poorer HRQoL when compared to the perception of adolescents as scores of physical, school and psychosocial dimensions reported by parents were 62.59 ± 26.47, 57.40 ± 21.85 and 68.81 ± 17.03, respectively. Table 3 shows that there were significant differences in mean scores for all dimensions of PedsQL™ 4.0 between adolescent self-report and parent proxy-report, except for the emotional functioning dimension. The mean total HRQoL scores reported by adolescents for both lower and higher-order scales of the PedsQL™ 4.0 were greater than 70, indicating good HRQoL [12] whereby they perceived themselves as being physically active and able to carry out normal daily activities. The mean total HRQoL scores reported by parents were 65.55 ± 18.96. This indicates adolescents perceived themselves to have a better HRQoL when compared to the perception of their parents.

**Table 3:** **Mean, standard deviation of the adolescent self-report and parent-proxy report**

**Dimensions**	**Mean ± SD**		
	**Adolescent-self report (n = 379)**	**Parent-proxy report (n = 218)**	**t-value**
**Lower-order of PedsQL™** **4.0**			
Physical function	78.97 ± 19.33	62.59 ± 26.47	7.51**
Emotional function	71.57 ± 22.94	71.24 ± 20.09	1.33
Social function	82.57 ± 20.08	75.50 ± 19.43	3.42**
School function	70.70 ± 19.14	57.40 ± 21.85	7.77**
**Higher-order of PedsQL™** **4.0**			
Physical function	78.97 ± 19.33	62.59 ± 26.47	7.51**
Psychosocial function	74.95 ± 17.65	68.81 ± 17.03	3.63**
Total HRQoL score	76.35 ± 16.66	65.55 ± 18.96	5.92**

**p < 0.01.

When compared by dimensions between the male and female adolescents, there was a significant difference in the emotional and social functioning whereby the mean emotional functioning scores were 69.52 ± 22.63 and 65.27 ± 21.29 for the male and female adolescents respectively (t = 3.120, p < 0.01) (Table 4). The mean social functioning score was 77.00 ± 22.51 and 82.77 ± 17.12 among the male and female adolescents respectively (t = 3.254, p < 0.01). However, there were no differences in the mean physical and school functioning score between the male and female adolescents. Moreover, there was no difference in mean psychosocial functioning score and total HRQoL score between the male and female adolescents.

**Table 4:** **Mean HRQoL scores by male and female adolescents**

**Dimensions**	**Mean ± SD**		
	**Male scores (n = 191)**	**Female scores (n = 188)**	**t-value**
**Lower-order of PedsQL™** **4.0**			
Physical function	77.42 ± 20.93	78.74 ± 16.57	0.789
Emotional function	69.52 ± 22.63	65.27 ± 21.29	3.120**
Social function	77.00 ± 22.51	82.77 ± 17.12	3.254**
School function	69.67 ± 20.75	71.90 ± 18.40	1.271
**Higher-order of PedsQL™** **4.0**			
Physical function	77.42 ± 20.93	78.74 ± 16.57	0.789
Psychosocial function	72.07 ± 19.20	73.31 ± 15.67	0.801
Total HRQoL score	73.40 ± 18.48	74.67 ± 14.60	0.858

**p < 0.01.

Further, there was no significant association between BMI-for-age (z-score), HRQoL dimensions and total HRQoL score of the adolescent self-report. On the other hand, there was a significant negative weak relationship between social functioning, psychosocial health dimension and total HRQoL score reported by parents with BMI-for-age (z-score) (r = -0.162, p < 0.05; r = -0.149, p < 0.05; r = -0.152, p = <0.05) respectively (Table 5).

**Table 5:** **Correlations between BMI-for-age and dimensions in PedsQL 4.0 using adolescent self-report and parent proxy-report**

**Dimensions**	**BMI-for-age (z-score)**	
	**Adolescent self-report**	**Parent proxy-report**
**Lower order of PedsQL™** **4.0**		
Physical function	0.034	-0.131
Emotional function	-0.065	-0.113
Social function	-0.014	-0.162*
School function	0.040	-0.092
**Higher order of PedsQL™** **4.0**		
Physical health	0.034	-0.131
Psychosocial health	-0.019	-0.149*
Total HRQoL score	0.011	-0.152*

*p < 0.05.

## Discussion

This current study examined the validity and reliability of the PedsQL™ 4.0 Generic Core Scales in adolescent self-report and parent proxy-report. Both adolescent self-report and parent proxy-report of PedsQL™ 4.0 showed convergent validity as the lower and higher-order scale of both these reports have high AVE (>0.5), factor loading and CR of more than 0.5. These results are in agreement with previous studies [18,20] whereby the results of CFA supported the existence of lower and higher-order scales as well as the convergent validity of this instrument.

When taking the results of fit-indices into consideration (CMIN/df, CFI, NFI, TLI, RMSEA) to test model fit, the lower and higher-order scales of the PedsQL™ 4.0 showed a range of average to good data fit for both adolescent self-report and parent proxy-report. This result supports the findings from a study by Petersen et al. (2009) who have reported that the PedsQL™ 4.0 met the cut-off criteria of the fit indices indicating an acceptable model fit [20,30]. Discriminant validity was also proven in both adolescent self-report and parent proxy-report as AVE values for the dimension were compared against the correlations between the factors and AVE, and they were found to be larger than the R^2^ of the correlations [18]. This study also reports acceptable levels of internal consistency reliability which is in line with the school samples from the United States, Norway, United Kingdom, Greece and Japan [8,31-34].

In this study, parents perceived HRQoL of their children as being poorer than the perception of the adolescents themselves. Parents scored lower in both the lower and higher-order scales of PedsQL™ 4.0 when compared to the scores by the adolescents, indicating parents have a different perspective on their children’s HRQoL. Previous studies also reported that HRQoL scores by parent proxy-reports have been consistently lower than the adolescent self-report [16,18]. The different perspectives of the parents on their children’s HRQoL could be due to the limited understanding of their children’s lives as well as their psychosocial and physical functioning [35]. On the other hand, parents may have an extensive perspective and broader point of view of their children’s overall HRQoL as parents are able to compare their children’s HRQoL to other adolescents [36,37]. Nevertheless, the perception of parents on their child’-ren’s HRQoL is important as parents are generally the ones who make decisions on health care and service utilization [11].

Although this study reported no difference in overall HRQoL by sex, adolescent males were found to have better emotional functioning than adolescent females. This result is in line with many studies as female adolescents have reported poorer HRQoL, specifically in the physical and emotional functioning dimensions when compared to the male adolescents [32,38-40]. Poor emotional functioning in female adolescents may be due to peer influence such as bullying and teasing due to physical appearance which could influence the individual’s self-esteem resulting in poorer HRQoL, particularly in the emotional functioning dimension [21]. Sociocultural pressures such as peer and family influences may also directly have a negative impact on the HRQoL of the adolescent girls resulting in poor overall HRQoL [21].

The prevalence of overweight and obesity among adolescents in the district of Kajang and Bangi has almost doubled from 19.5% [41] to 25.8% as reported in the current study. The prevalence of overweight and obesity was almost similar or higher compared to other studies conducted among Malaysian adolescents [42-44]. This could be explained as Malaysia is undergoing a transitional period of nutrition and lifestyle changes due to industrialization, urbanization and globalization.

Since the overweight and obesity problem is one of the most important factors contributing to poor HRQoL among adolescents, the current study assessed the correlation between BMI-for-age and HRQoL. Parent proxy-report of the PedsQL™ 4.0 showed a significant inverse association between total HRQoL score, social functioning, psychosocial health with BMI-for-age. This indicates that as BMI-for-age of the adolescents’ increases, the total HRQoL score by the parent proxy-report decreases. The contradicting findings between the reports could be due to the different perceptions of HRQoL among the adolescents and their parents [31,36]. Further, parents may have uncertain feelings on several aspects related to adolescent HRQoL as they may not spend much time with their children [45,46]. Parents may also be able to observe the overall HRQoL of their children from an external point of view as an outsider when compared to the adolescent himself [45]. Therefore, parental perception of adolescents’ HRQoL should also be taken into consideration. However, adolescent self-report did not show any significant relationship towards BMI-for-age. The non-significant relationship between BMI-for-age and HRQoL in the adolescent self-report can be explained by the non-linear result between BMI-for-age and HRQoL. Other adolescents who are not overweight and obese may also have poor HRQoL mainly due to certain factors such as malnutrition which could cause limitations in the physical functioning [40].

Our study has several limitations that should be taken into consideration. Firstly, the participants involved in this study were 13 to 17 years old, thus this study is not applicable to children or early adolescents. Future studies are recommended to involve young adolescents since the PedsQL™ 4.0 is valid to be used among adolescents (10-19 years) as defined by WHO [16]. Secondly, the temporal relationship could not be established due to the nature of a cross-sectional study design. Therefore, the cause and effect relationship between BMI and HRQoL could not be determined. Furthermore, other type of validity and reliability tests such as criterion validity and test-retest reliability were not determined in this study. Hence, future studies should include these validity and reliability tests to further determine the validity and reliability of the PedsQL™ 4.0.

## Conclusion

This study demonstrates that adolescent self-report and parent proxy-report of PedsQL™ 4.0 Generic Core Scales are valid and reliable to assess the HRQoL of Malaysian adolescents in a non–clinical population. Therefore, it would be recommended to use both the adolescent self-report and parent proxy-report of PedsQL™ 4.0 in future studies. The parent proxy-report of PedsQL™ 4.0 is able to provide a different perspective on the HRQoL of their children.

## Abbreviations

HRQoL: Health-related quality of life
PedsQL™: Pediatric quality of Life inventory™
CMIN/df: Chi-square/degrees of freedom
CFA: Confirmatory factor analysis
CFI: Comparative fit index
NFI: Normed fit index
TLI: Tucker lewis index
AVE: Average variance extracted
RMSEA: Root mean squared error of approximation
